# A Study on the Fracture of Brittle Heterogeneous Materials Using Non-Extensive Statistical Mechanics and the Energy Distribution Function

**DOI:** 10.3390/ma18020335

**Published:** 2025-01-13

**Authors:** Dimos Triantis, Ilias Stavrakas, Ermioni D. Pasiou, Stavros K. Kourkoulis

**Affiliations:** 1 Electronic Devices and Materials Laboratory, Department of Electrical and Electronics Engineering, Faculty of Engineering, University of West Attica, Ancient Olive Grove Campus, Building B, 250 Thivon Avenue, 122 44 Athens, Greece; triantis@uniwa.gr (D.T.); ilias@uniwa.gr (I.S.); 2 Laboratory for Testing and Materials, Department of Mechanics, School of Applied Mathematical and Physical Sciences, National Technical University of Athens, Zografou Campus, 157 73 Athens, Greece; epasiou@central.ntua.gr

**Keywords:** non-extensive statistical mechanics, Tsallis entropy, entropic index, acoustic emissions, energy distribution function

## Abstract

The fracture process of heterogeneous materials is studied here in the framework of the discipline of Non-Extensive Statistical Mechanics. Acoustic emission data provided by an experimental protocol with concrete specimens, plain or fiber-reinforced, under bending are taken advantage of. This innovation of the study lies in the fact that the analysis of the acoustic activity is implemented in terms of the energy content of the acoustic signals rather than of their interevent time or their interevent distance. The Energy Distribution Functions were properly fitted using the expression proposed by Shcherbakov, Kuksenko and Chmelet. This study reveals that the loading and fracture processes of the specific materials are definitely non-additive and non-extensive. It is concluded that the presence of notches is crucial since it assigns non-additivity and non-extensivity from relatively low loading levels due to the early formation of the fracture process zone around the crown of the notch. The values of the Tsallis entropic index, q, that were determined are in very good agreement with the respective ones obtained in previous studies by means of different analysis tools. Finally, a clear correlation between the index q and the average energy content of the acoustic signals is highlighted for the whole range of values of the energy content of the acoustic signals.

## 1. Introduction

It is well known that while brittle heterogeneous materials are loaded mechanically at load levels causing damage, strain energy that is stored within the mass of the material is released, in the form of elastic waves, due to sudden stress distributions. These elastic waves propagate through the mass of the loaded body, and upon reaching the external surfaces of the body, they can be detected and stored as electric signals using proper sensors. The sensing technique based on the proper elaboration of these signals is widely known as the acoustic emission (AE) technique and nowadays is the most mature and well-founded Structural Health Monitoring (SHM) tool [1,2,3]. This provides, among others, valuable information about the damage mechanisms that are activated within the bulk of a loaded structure (the generation and propagation of micro-cracks, their mutual interaction and coalescence in the direction of forming macro-cracks that will, finally, lead to the failure of the loaded element) [4], also permitting estimations about the remaining load-carrying capacity of the structure or the structural element [5,6]. Especially for structures made of concrete (the material that will be studied in the present protocol), monitoring damage using the AE technique is at present a sine qua non for safe conclusions to be drawn concerning the “health” of a structure and its proximity to catastrophic fracture [7,8,9,10].

From the thermodynamic point of view, fracture is a highly non-equilibrium process in open systems. Especially for brittle materials (like, for example, rock and concrete), the activation of damage mechanisms results in the formation of “fractal structures” within the bulk of a loaded system, leading, in turn, to self-organized critical states [6,11,12,13,14,15]. Indeed, a long series of experimental protocols, with a large variety of specimens’ geometries and various loading schemes, have highlighted that as the applied load tends to its critical limits, the rate of generation of acoustic events and counts increases strongly and systematically [16,17,18,19]. Moreover, for protocols comprising compressive loading, the temporal evolution of the energy content of the acoustic events can be described by specific power laws [16,20]. Proper elaboration of the information provided by the acoustic signals is achieved by means of either traditional analysis tools or innovative ones based on machine learning [21,22,23]. The effective use of the latter is greatly assisted by the development of Artificial Intelligence. Very interesting and pioneering related studies have been published that attempt to safely assess the damage level and, also, to quantify the remaining life of heterogeneous structural materials [24,25,26,27].

Taking into account that the generation of acoustic events due to micro-cracking and stress redistribution at the interior of a loaded system exhibits strong similarities with the processes generating earthquakes, efforts are recorded worldwide to analyze laboratory-recorded acoustic emissions using tools that were initially introduced for the analysis of seismic events (assuming of course that these tools are properly adapted to the scale and to the specific features of the laboratory experiments [28,29,30,31]).

In this direction, it should always be kept in mind that the fracture processes (either observed at laboratory or earthquake scales) are manifestations of non-linear processes, which characterize complex dynamical systems (obviously at quite different scales), and, therefore, their study demands advanced statistical tools rather than the traditional tools of classical Statistical Mechanics [32,33,34]. However, for complex dynamical systems, which are at a stage of non-equilibrium and exhibit evolution governed by external factors (for example, externally imposed mechanical loads), the familiar thermodynamic principle of additivity is often proven inadequate for the proper analysis and understanding of the temporal evolution of the system under study. This inadequacy is well attributed to the non-independent nature of the sources of the acoustic emissions, namely of the micro-cracking events, which, beyond specific load levels, are gradually organized in the direction of forming mutually interacting networks of micro-cracks. In this context, the concepts of Boltzmann–Gibbs (BG) Statistical Mechanics should be properly modified in order to cope with phenomena that are characterized by long-range interactions (and, perhaps, memory effects), giving birth to the discipline of Non-Extensive Statistical Mechanics (NESM). At present, the most widely accepted formulation of NESM is the one proposed by Tsallis [35], which is based on an alternative definition of entropy (usually denoted as “Tsallis entropy”) for complex systems in non-equilibrium conditions as follows:

(1) $$S_{q}=\frac{k\left(1-\sum_{i=1}^{w}P_{i}^{q}\right)}{q-1}=1$$

In Equation (1), k is Boltzmann’s constant, P_i_ is the probability of appearing of the ith configuration, w is the number of possible configurations and q is the so-called entropic index.

It is nowadays proven that Tsallis entropy, S_q_, describes adequately complex dynamical systems in a long series of scientific disciplines ranging from Physics and Astronomy to Mechanics of Materials and Social and Financial Sciences [36,37]. The entropic index q quantifies the degree of non-additivity of the processes taking place. It is, in fact, a measure of the non-extensivity of the system (it is here recalled that the concept of non-additivity is not identical to the concept of non-extensivity). Obviously, in the q→1 limit, Tsallis entropy tends to that of BG Statistical Mechanics. On the other hand, values of q > 1 (the case which is of importance in the present study) imply systems composed of sub-systems characterized by long-range mutual interactions. Finally, when q < 1, the system is in equilibrium with a relatively limited number of events. It is, also, considered that q quantifies the extent of mutual interactions: The higher the values of q, the longer the range of mutual interactions. Especially for the disciplines of Mechanics of Materials and Structural Health Monitoring, it is accepted that the Tsallis approach to NESM offers a quite flexible and effective tool for the description, analysis and understanding of the sequence of the damage processes within a mechanically loaded system.

Recalling now that (i) the fracturing of heterogeneous materials is a critical phenomenon of complex dynamical systems [38,39] and, also, that (ii) the entropic index q is a measure of the deviation of a loaded complex system from extensivity, the present study aims to quantify the temporal evolution of this index (during three-point bending of beam-shaped concrete specimens) from the very early loading steps up to the instant of macroscopic failure. The final target of this study is to correlate the evolution of q with the respective damage mechanisms activated, leading finally to fracture. This target is achieved by exploiting experimental data provided by the acoustic emission technique. The innovative aspect of this study is that the parameter used is related to the energy content of the acoustic signals rather than to the interevent time intervals (i.e., the interval between any two successive acoustic signals) or the interevent distances [38,39].

The approach adopted here vividly enlightens the phenomena that take place during the very last loading steps (in other words, the instants at which the system is about to enter into the critical stage of impending catastrophic propagation of the fatal macro-crack), firmly underlying that the loading and fracture processes of the heterogeneous materials studied are definitely non-additive and non-extensive. In addition, it is revealed that, in the presence of pre-existing notches, non-extensivity and non-additivity characterize the response of the loaded materials from very early loading levels. Finally, the present study indicates that by analyzing the temporal evolution of the q-index in terms of the energy content of the acoustic hits, signs are detected, which can be considered as “pre-failure indices”, i.e., signs of early warning about upcoming disastrous failure.

## 2. Materials and Methods

### 2.1. Theoretical Preliminaries

In the frame of the Tsallis formulation of NESM, Sotolongo-Costa and Posadas [40] introduced an empirical model adequately describing the distribution of the magnitude of seismic events. The specific model was then developed further by Silva et al. [41] and, also, by Telesca [42]. The respective Cumulative Distribution Function for the energy (sometimes called the Energy Distribution Function—EDF) reads as follows:

(2) $$\log\left(N_{>m}\right)=\log N+\frac{2-q}{1-q}\log\left[1-\frac{1-q}{2-q}\left(\frac{10^{2m}}{\alpha^{2/3}}\right)\right]$$

In the above equation, N_>m_ is the number of seismic events with a magnitude that exceeds a predefined threshold m. Moreover, N is the overall number of seismic events recorded, and α is a proportionality constant (proportionality between the energy of an event and the size of the fragment responsible for this event). Finally, q is the Tsallis entropic index (see Equation (1)).

Adopting the above-mentioned ideas, Shcherbakov et al. [43], in 2011, published a pioneering paper, modifying the model introduced by Equation (2), in order to describe the distribution of the energy that is released during micro-cracking for systems (specimens) loaded mechanically at levels approaching those causing macroscopic fracture. This modified Energy Distribution Function reads as follows:

(3) $$N\left(E>E_{i}\right)=N_{t}{\left[1-\frac{1-q}{2-q}{\left(\frac{E_{i}}{\alpha }\right)}^{\frac{2}{3}}\right]}^{\frac{2-q}{1-q}}$$

In Equation (3), N(E > E_i_) represents the number of acoustic events (recorded during a loading and fracture process) with an energy content, E, that exceeds the predefined threshold E_i_. Moreover, N_t_ corresponds to the total number of acoustic events recorded during the loading process. Finally, parameter α denotes the energy density, and q is the Tsallis entropic index. Following Shcherbakov et al. [43], the parameters α and q are determined by demanding optimum agreement between experimental data and analytical results.

Now, adopting the above argumentation, an attempt is undertaken in this study to quantify the degree of thermodynamic non-equilibrium and non-additivity during the various stages of mechanical loading of brittle heterogeneous building materials. In this effort, the temporal evolution of the energy content of the acoustic hits recorded during an experimental protocol with concrete beams loaded according to a quasi-static, three-point bending, loading scheme is taken advantage of. The target of the present study is to investigate whether the distribution of the energy content of the acoustic hits recorded at the stages of generation of micro-cracks, of their growing (in the direction of forming mutually interacting networks) and, finally, of their mutual coalescence (in the direction of forming fatal macro-cracks), could be effectively described in terms of the Tsallis formulation of NESM using Equation (3).

The present attempt is triggered by the conclusions of a series of previous studies which verified that the Cumulative Distribution Function of the interevent time intervals, δτ, between any two successive acoustic events is indeed described by a q-exponential function. More specifically, Vallianatos et al. [39] proved that, while basalt specimens are uniaxially compressed until fracture, the distribution of both the interevent time intervals and the Euclidean interevent distances obeys a specific q-exponential function. Similar conclusions were drawn by Vinciguerra et al. [44] based on experimental protocols with specimens made of granite and sandstone and, also, by Saltas et al. [45] and Loukidis et al. [46], who used specimens made of marble. Especially for protocols with specimens made of marble (either under monotonic loading or under loading schemes comprising stepped-stress increments), it was definitely highlighted that, as the applied load tends to that causing fracture, the entropic index q increases smoothly towards a value equal to about q ≈ 1.5. Then, very shortly before the fatal macroscopic cracking, q decreases rapidly [47], providing an interesting, at least, pre-failure index. Similar approaches were reported by Greco et al. [48] in their effort to describe the distribution of the interevent time intervals in protocols with specimens made of basalt submitted to cyclic compression. The variability of q during various stages of the loading process was definitely confirmed, as early as 2013, by Stergiopoulos et al. [49] in protocols with cement mortar beams, and it was reconsidered very recently by Naukhez et al. [50], who used specimens made of a variety of ultra-high-performance concrete.

### 2.2. The Experimental Protocol and the Mechanical Response of the Specimens

Within the above-described framework, the present study will take advantage of acoustic emission data that were recorded during the stages of the generation, development and coalescence of micro-cracks, in an attempt to approximate the EDF of the acoustic hits in terms of Equation (3), in order to quantify the parameters q and α and determine their possible dependence on the specific composition of the materials tested. The experimental protocol of the present study has been described analytically in a previous paper by the authors’ team [51], and, therefore, it is only very shortly outlined here.

**Figure 1:**
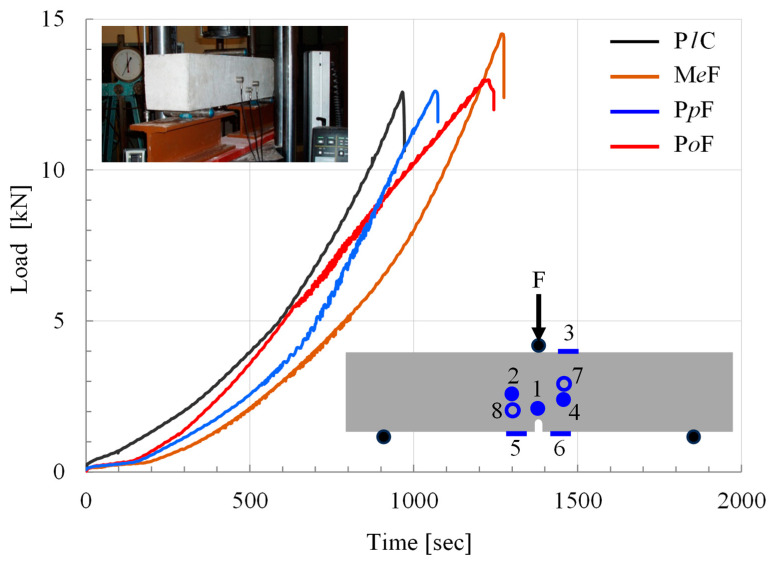
The temporal evolution of the load applied for typical specimens of the four classes of beams tested. The lower embedded sketch schematically describes the geometry of the specimens and the position of the acoustic sensors (empty circles indicate the sensors positioned at the rear vertical face of the beams), which are numbered from 1 to 8. The upper embedded photo shows a P*l*C-class specimen during testing.

Beam-shaped specimens, of length equal to 750 mm and of square cross-section (150 × 150 mm^2^), were tested. All specimens were mechanically notched at their mid-span. The notches were machined at a depth of 25 mm, and their width was equal to 5 mm. The specimens were submitted to three-point bending with the aid of a stiff Instron-Satec servo-hydraulic loading frame with a capacity equal to 300 kN. The specimens were supported using two steel cylinders, at a distance from each other equal to 600 mm on either side of the notch, as can be seen schematically in the draft sketch embedded in Figure 1. The load was applied monotonically up to the fracture of the specimens with the aid of a third steel cylinder placed on the notched section of the beams. Displacement-control conditions were adopted at a rate equal to 0.08 mm/min. Four classes of specimens were tested: a class including beams made of plain concrete (denoted from here on as P*l*C class) and three classes including beams made of fiber-reinforced concrete. The reinforcing fibers were relatively short, and they were made of steel (M*e*F class), Polypropylene (P*p*F class) or Polyolefin (P*o*F class). The characteristics of the fibers and the composition of the specimens of each class are listed in ref. [51].

Three to four specimens were tested from each class, and their mechanical response was characterized by small discrepancies between specimens of the same class (see Figure 1). The same is true for the acoustic activity recorded. In this context, in the analysis following, one representative experiment is discussed from each class. It could be anticipated that the number of specimens is small for systematic statistical analysis to be carried out. In any case, this is the first attempt to cope with the specific issue (quantitative description of the temporal evolution of the entropic index by means of the energy content of the acoustic hits), and it is obvious that additional experimental protocols, with a wider variety of materials and loading protocols, are required before definite conclusions are drawn. However, the small discrepancies observed between identical specimens and, above all, the positive correlation of the outcomes of this study with those of earlier ones (which confronted the problem from a completely different point of view, as will be outlined in the Section 4) are, at least, encouraging and promising hints.

The acoustic activity was detected using eight acoustic sensors (properly attached at strategic points of the beams) of the R15α type, provided by Mistras Group, Inc–Physical Acoustics Corp., West Windsor Township, NJ, USA. This is a general-purpose sensor with a resonant frequency of 150kHz. Its effective bandwidth lies in the region between 50 kHz and 400 kHz [52], which is a region fully compatible with the material tested in the present protocol. Its resonant frequency, (Ref V/(m/s)) is 75 kHz, and its peak sensitivity (Ref V/(m/s)) is equal to 80 dB. The sensors are controlled by the PCI-2 hardware, a low-noise digital signal processing system with an 18-bit A/D converter, capable of attaining sampling rates equal to 40 Msample/s [52].

The threshold of the amplitude of the acoustic signals was set to 40 dB. This value is generally used in the respective literature as a proper limit, ensuring that no surrounding noise will be recorded. In the present protocol, the AE ASL (Average Signal Level), namely, the quantity that actually reflects the level of the surrounding AE activity, was recorded constantly. It was noticed that it never exceeded the value of 25 dB. It is exactly this gap between the maximum ASL of 25 dB and the 40 dB threshold that guarantees that the recorded data are of high quality, concerning their purity from external noises. Careful selection of the AE timing parameters was, also, a crucial parameter ensuring the quality of the recorded AE data. The vendor provides a set of processes to ensure proper estimation of the timing parameters, like Peak Definition Time (PDT), Hit Lockout Time (HLT) and Hit Definition Time (HDT). Following the suggestions of the vendor, it was concluded that the most proper values of the above parameters were as follows: PDT: 200 ms, HDT: 400 ms, HLT: 100 ms. In order to check the appropriateness of the values adopted, series of preliminary tests were performed with dummy specimens. It was ensured that the AE hits were distinguishable and not overlapped or under-sampled [53].

For the needs of this study, use was made of the acoustic hits detected by the sensor located at the crown of the notch (Sensor 1 in the sketch embedded in Figure 1). The main reason for this choice is that the presence of the notch strongly amplifies the stress field in the immediate vicinity of its crown, and, therefore, Sensor 1 collects the most representative and strong hits from the location at which damages are expected to cause the initiation of the macroscopic crack. In addition, the hits recorded by this sensor are the ones most clear from noises due to rebound waves.

The main characteristic of the mechanical response of the specimens was that only the ones of the P*l*C class were actually fragmented into two pieces (almost at the instant that the applied load attained its maximum value). The specimens of the remaining three classes were not fragmented at the peak load. For these specimens, at the instant of load maximization, a macroscopically visible crack started propagating very slowly (from the crown of the notch towards the load application cylinder) while the applied load exhibited an abrupt drop to a level equal to about one-third of the maximum value attained. Obviously, after this abrupt load drop and the onset of propagation of the catastrophic macro-crack front, the load-carrying capacity of the specimen is, in fact, diminished (the two pieces of the beams are just kept together due to the action of the reinforcing fibers located in the vicinity of the central cross-section). In this context, the analysis of the experimental data recorded by the acoustic sensor is terminated at the instant of the abrupt load drop for all four classes of specimens. The mechanical response of typical specimens from each class of beams is shown in Figure 1, in which the temporal evolution of the load applied is plotted. The color code used to distinguish between the specimens of the four classes will be used throughout the analysis following.

## 3. Results

### 3.1. Temporal Evolution of the Energy Content of the Acoustic Hits

As a first step, the energy content of the acoustic hits recorded during the overall duration of four representative experiments (one from each class) was considered in juxtaposition to the applied load. It was decided to plot the values of the energy content of the acoustic hits (and those of the applied load) versus the time-to-failure, t_f_-t, parameter, where t_f_ is the instant of fracture (which is, in fact, the instant of the abrupt load drop). The specific parameter, especially if it is plotted along a logarithmic scale, permits a kind of “magnified view” into the time interval close to the terminal instant of the experiment (the fracture instant). Therefore, it provides a clearer insight into the events that take place during the very last instants before the maximization of the load applied and the onset of macroscopic fracture. This is quite crucial for the analysis of the acoustic activity since the vast majority of acoustic hits are recorded during the last few seconds before the onset of propagation of the fatal macro-crack front.

**Figure 2:**
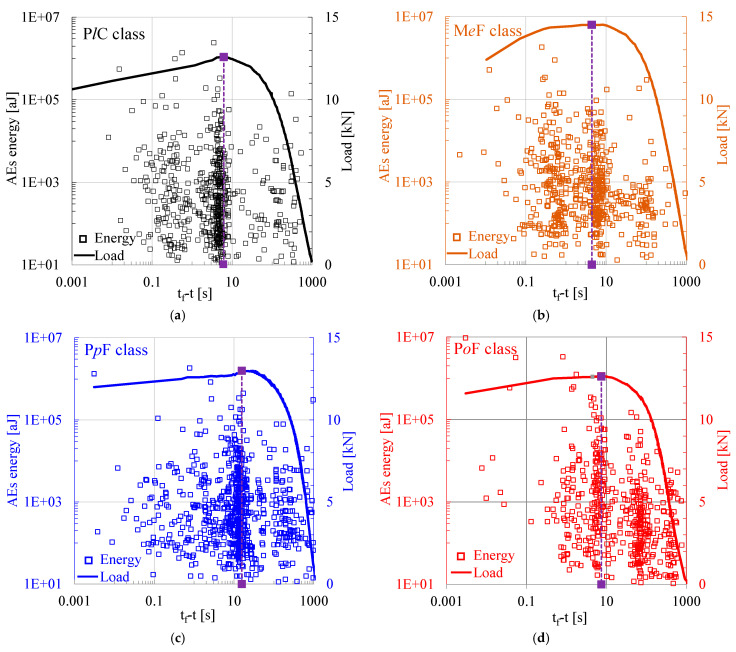
The energy content of the acoustic hits recorded during four typical experiments (one from each class) in juxtaposition to the temporal evolution of the applied load vs. the time to failure (t_f_-t) parameter, adopting logarithmic scales for both axes: (**a**) P*l*C class; (**b**) M*e*F class; (**c**) P*p*F class; (**d**) P*o*F class.

The outcomes of the above-described procedure are shown in Figure 2 (in which the time instant of maximization of the applied load, t*, is indicated with the vertical purple dashed line for all four experiments that are discussed in this section). It is clearly seen from this figure that the instant at which the load is maximized slightly precedes the instant of abrupt load drop. The difference between these two critical instants ranges from about 4 s to about 15 s. In addition, it is seen that the energy content of the acoustic hits varies, approximately, in the interval from 10 aJ to 10 pJ, with relatively minor discrepancies between the four classes of specimens.

Numerical values of some critical parameters are recapitulated in Table 1. These parameters include the above-mentioned critical time instants t* and t_f_, the maximum load attained L_max_, the total number N_t_ of the acoustic hits recorded up to the fracture (abrupt load drop) of the specimen, and the number of acoustic hits recorded in the time intervals t ≤ t* and t* < t ≤ t_f_, which are denoted as N_1_ and N_2_, respectively.

### 3.2. The Energy Distribution Function of the Acoustic Hits and the Variation in the Entropic Index

**Figure 3:**
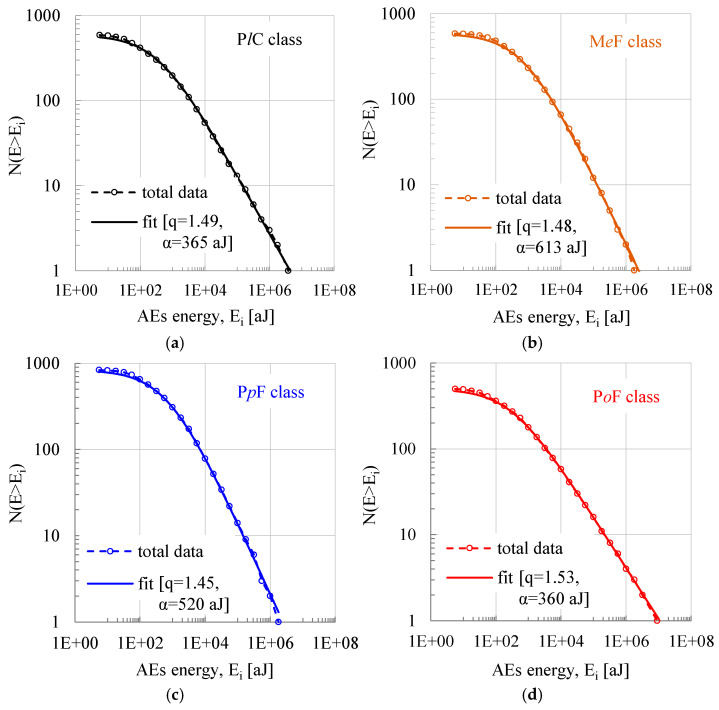
The Cumulative Distribution Function for the energy content of the acoustic hits. Experimental data are denoted by empty markers while the curves fitted to the data by means of Equation (3) are denoted by continuous lines. (**a**) P*l*C class; (**b**) M*e*F class; (**c**) P*p*F class; (**d**) P*o*F class.

As a next step, the Cumulative Distribution Function of the energy content of the acoustic hits (Energy Distribution Function—EDF) is plotted in Figure 3 (taking into account the overall number of hits recorded), for the four specimens discussed here. The experimental data (empty circular markers) are then fitted by means of Equation (3) (continuous line) by properly choosing the numerical values of the parameter α and the entropic index q, in order to achieve optimum fitting. The fitting procedure by means of Equation (3) was of excellent quality: in all cases (without any exception), the coefficient of determination R^2^ ranged in the interval 0.98 < R^2^ < 1.0. The numerical values of α and q are depicted in each one of the plots in Figure 3.

It is easily concluded from Figure 3 that Equation (3) excellently describes the distribution of the energy content of the acoustic hits for the whole range of the energy values for all four classes of specimens. It is seen, also, that the values of q vary in a very narrow interval (1.45 < q < 1.53), while those of the parameter α vary in the interval 360 aJ < α < 613 aJ.

It is very interesting to highlight at this point that values of the entropic index close to q ≈ 1.5 are quite often reported in the literature for protocols dealing with mechanical systems (specimens) loaded by external stimuli leading to macroscopic fracture. Indicatively only, one could mention the relatively recent paper by Sychev and Kulkov [54]. They studied the response of specimens made of sandstone, marble and granite using the Energy Distribution Function of the acoustic signals. For monotonic compressive loading schemes leading to fracture, they arrived at the conclusion that the processes are governed by the concepts of Tsallis NESM with values of q equal to about q ≈ 1.5. Similarly, in a recently published paper, Burud and Chandra Kishen [55], while studying the acoustic emissions in specimens made of concrete-like disordered materials in the frame of Tsallis NESM using a power-function approach, arrived at a value of q equal to q = 1.54.

What is, perhaps, more striking is that quite similar values for the entropic index q were obtained by considering the Cumulative Distribution Function of a different critical parameter related to the acoustic activity, namely, the interevent time interval (the interval between any two successive acoustic signals). Indeed, Loukidis et al. [47] concluded that while compressing uniaxially intact prismatic marble specimens (either monotonically or by means of stepped-stress increment loading schemes), the entropic index, at the last loading stages (i.e., while the system is about to enter into its critical stage), attains values equal to about q ≈ 1.5. Similar values (q ≈ 1.48) were obtained by Vinciguerra et al. [44] for granite specimens and by Saltas et al. [45] for sandstone specimens. For specimens made of ultra-high-performance concrete with steel fibers and coarse aggregate under bending, Stergiopoulos et al. [49] arrived at values for the entropic index equal to about q ≈ 1.47. Stavrakas et al. [56], while submitting cement mortar specimens to bending under a cyclic loading protocol, determined q-values equal to q ≈ 1.42 in the last loading cycle. In addition, while implementing structural tests with specimens of large dimensions (simulating restored structural elements of ancient monuments) under pure shear, Kourkoulis et al. [57] calculated values for q in the 1.51 < q < 1.58 interval.

**Figure 4:**
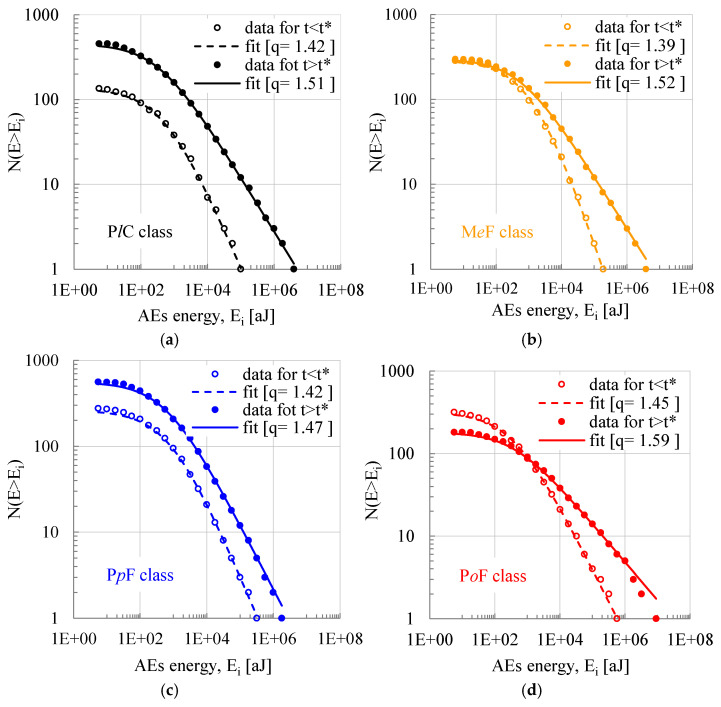
The Cumulative Distribution Function for the energy content of the acoustic hits recorded during the four typical experiments considered. Empty circular markers and dotted lines represent the acoustic hits recorded during the interval t < t* (Group I), while filled markers and continuous lines represent the acoustic hits recorded during the interval t* < t < t_f_ (Group II). (**a**) P*l*C class; (**b**) M*e*F class; (**c**) P*p*F class; (**d**) P*o*F class.

At this stage of the analysis, it was deemed interesting to study the Cumulative Distribution Function of the energy content of the acoustic hits by dividing them into two groups: In the first one (Group I), the acoustic hits recorded before the instant of load maximization (i.e., the instant t*) were included. In the second one (Group II), the acoustic hits recorded in the interval t* < t < t_f_ (i.e., between the instant of load maximization and that of fracture) were included. In this interval, the load decreases smoothly and almost imperceptibly (it is recalled again that the load maximization instant precedes that of macroscopic fracture). The fitting procedure was conducted for each group separately, and the corresponding results are presented in Figure 4, together with the values of q that provide optimum fitting in terms of Equation (3). It is interesting to observe that, for all classes of specimens, acoustic hits with high energy content prevail after the instant of load maximization. Equally important is the fact that the values of the entropic index q, fitting the experimental data for the Energy Distribution Function according to Equation (3) in the 0 < t < t* interval, are systematically lower compared to the respective values of q for the t* < t < t_f_ interval. Moreover, the values of q best fitting the whole range of values (i.e., for the interval 0 < t < t_f_) are, also, lower compared to the respective ones recorded in the t* < t < t_f_ interval. The above observations clearly support the statement that as the system (specimen) proceeds towards its critical stage (namely, the stage of impending catastrophic fracture), the level of organization of the sub-systems characterizing the damage processes within the mass of the system (i.e., the networks of micro-cracks) keeps increasing, and the system is gradually “moving” away from the thermodynamic conditions characterizing systems and processes for which additivity and extensivity prevail. A synopsis of the numerical values of the most important quantities of the above discussion is recapitulated in Table 2.

**Figure 5:**
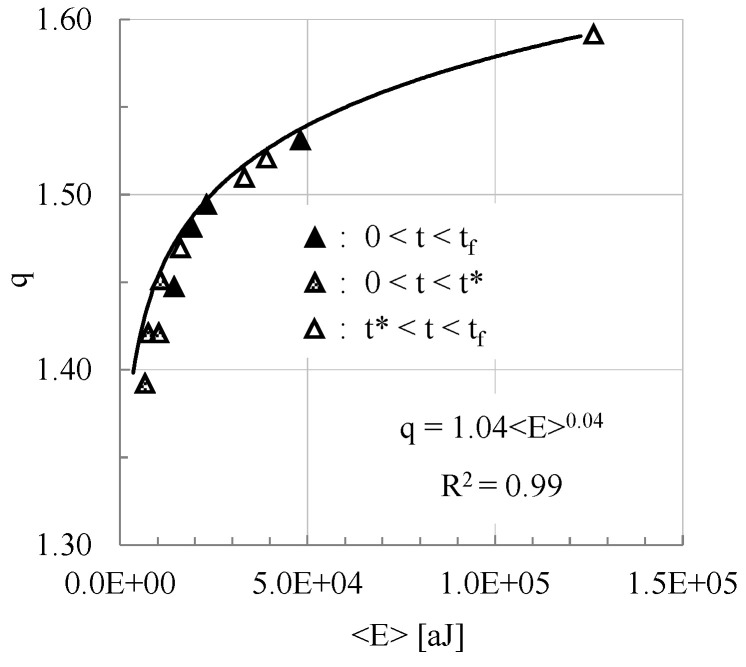
The dependence of the entropic index q on the average energy content of the acoustic hits, for the overall duration of the four tests analyzed and, also, for the stages before and after the maximization of the applied load.

A more comprehensive overview of the above findings is provided in Figure 5, in which the correlation between the numerical values of the entropic index, q, and those of the average energy of the respective distribution is more evident. Based on this figure, it is concluded that the dependence of the entropic index on the respective average energy content of the acoustic hits is governed by a power law of the form q = A < E > ^C^, where A and C are numerical constants obtained by means of optimum fitting. It is, also, interesting to observe that both stages of the loading process (i.e., that before load maximization and that after this instant up to the fracture of the specimens), as well as the overall loading process, are governed by the same law (i.e., a power law with the same A and C constants).

## 4. Discussion

The acoustic activity developed in prismatic beams made of concrete (either plain or fiber-reinforced) submitted to three-point bending was studied in the frame of NESM (Non-Extensive Statistical Mechanics), adopting the modified definition of entropy introduced by Tsallis. The above decisions were made taking into account that, at present, NESM is considered an appropriate framework for modeling the thermodynamics of complex systems and information of complex signals [6,11,12,13,14,58,59,60,61,62,63,64,65]. Moreover, its appropriateness for the description of the response of brittle heterogeneous materials, as well as for the quantification of the damage within their bulk, is discussed in a very long series of publications during the last thirty years. Indicatively, one could mention refs. [36,43,44,45,46,47,48,49,50,55,56,57,66,67]. For example, Shcherbakov et al. [43] mentioned characteristically that for studies in the field of fracture processes “…The recent appearance of and progress in non-extensive statistical mechanics based on Tsallis’ concept is a breakthrough in this field. This method allows one to analytically describe phenomena for which only experimental dependences were previously known”. Within the same frame, Vinciguerra et al. [44], while studying fracture and failure of two different materials (namely Darley Dale Sandstone and AG Granite) using the q-formalism, definitely concluded “…that pre-failure processes in both types of rocks, can be reproduced with q-exponential curves, showing universal features …”. From a completely different point of view, Shan et al. [67], who studied the electric emissions (in terms of the Electric Potential—EP) produced while loading brittle heterogeneous materials, concluded that the Probability Density Function of the Electric Potentials “… follows q-Gaussian distribution for all samples with q values greater than 1 and less than 2, which conforms to the non-extensive statistical characteristics”. Moreover, they clearly highlighted that “… The q value of EPs can be used as an effective prediction index like the b value of AEs”. Quite a few additional examples, justifying the choices made in this study, could be cited; however, for the sake of brevity, readers who are interested in additional information may refer to the above-mentioned long series of related papers [36,43,44,45,46,47,48,49,50,55,56,57,66,67].

As soon as the frame of NESM by means of Tsallis entropy was chosen, the choice of the q-index as the most appropriate parameter for the analysis was straightforward: It properly quantifies the degree of non-additivity of the loaded system, and, in addition, it is the most suitable parameter that provides indicators concerning the stability of loaded systems (and therefore their impending entrance into a critical stage) for a large variety of scales, appropriate for the study of laboratory specimens, structural elements and structures, and, also, the crust of the Earth (i.e., for the study of seismic events) [68]. As far as it concerns the choice of the energy content of the acoustic signals as the most suitable parameter for the quantification of non-additivity of the fracture processes, it was dictated by the scarcity of related studies. Indeed, the vast majority of related studies quantify non-additivity using either the interevent time (IT) or the interevent distance (ID). Obviously, if the conclusions drawn using the energy content of the acoustic signals are in accordance with those drawn using the IT or the ID, this would be additional support for the approach stating that for brittle heterogeneous materials, the traditional Boltzmann–Gibbs theory of Statistical Mechanics must be properly modified to cope with phenomena showing features of long-range correlations, long-term memory, and multiple fractals. It is mentioned, however, that the choice of alternative parameters would not alter the results from a qualitative point of view, although it could yield quantitative differences.

In any case, the above decisions and choices are in accordance with the contemporary trend (prevailing in the discipline of Strength of Materials) to move from the macroscopic description of fracture processes to the microscopic one, a trend greatly assisted by the rapid development of sensing tools that are capable of providing information from the interior of loaded structures. This trend has been proven very effective, especially in the case of disordered materials such as concrete and natural building materials, like marble and rocks. The reason is that, for such materials, the fracture process zone formed around crack-like defects is relatively extensive compared to other characteristic lengths of their structure. The damage processes within these extended fracture process zones (like, for example, mutually interacting networks of micro-cracks within the matrix of the cement, interactions between the micro-cracks and the aggregates, as well as between the micro-cracks and micropores) are quite complex, and, moreover, they are inevitably characterized by “…long-range (or short interactions with long-range effect) interactions among the constituents of the material, the inherent defects, the multi-scale cracks, and slow decaying stress field” [55]. Taking into account that the mutually interacting entities of these materials cannot be separated from each other, the whole system behaves as a “sum” of mutually interacting constituents (or, equivalently, of sub-systems), the response of which is inevitably characterized by non-additivity (and, also, by non-extensivity). The response of these systems cannot be effectively described using the traditional Boltzmann–Gibbs Statistical Mechanics and, in this way, resorting to Non-Extensive Statistical Mechanics approaches, like the one introduced by Tsallis, becomes a pressing demand.

The efficiency of these approaches in the description of phenomena in a wide variety of disciplines is, at present, accepted broadly. Especially in the discipline of Mechanics of Materials, these approaches usually take advantage of the Cumulative Distribution Function of the interevent time intervals (see, for example, refs. [41,42,43,44,45,46,47,48,49]) between any two successive acoustic signals. However, alternative approaches based on the Cumulative Distribution Function of the energy content of the acoustic hits were, also, introduced, as early as the beginning of the 21st century. These energy-based approaches were inspired by much older theories introduced in the discipline of Seismology.

In this study, the approach proposed by Shcherbakov et al. [43] (based on the Sotolongo-Costa and Posadas [40] analytical expression for the Cumulative Distribution Function of the magnitudes of seismic events) was adopted. The outcomes of this study definitely supported the idea that the Cumulative Distribution Function of the energy content of the acoustic signals recorded during the mechanical loading of concrete structures obeys a q-formalism, identical to that governing the respective function of the magnitudes of earthquakes. Moreover, it was concluded that the values of the entropic index increase with increasing load, suggesting that the loaded system exhibits a gradually increasing degree of non-additivity. The numerical values of q calculated varied in the 1.39 < q < 1.59 interval, either considering the loading process as a whole or dividing it into two sub-intervals.

**Figure 6:**
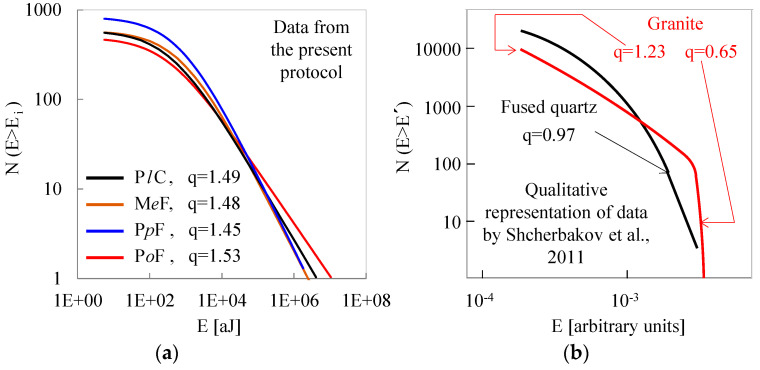
(**a**) The Cumulative Distribution Function for the energy content of the acoustic hits recorded during typical experiments of the present protocol; (**b**) qualitative representation of the data for the Cumulative Distribution Function for the energy, based on Figure 2 of the milestone paper by Shcherbakov et al. [43].

At this point, it is necessary to consider the findings of the present study in comparison with those provided by Shcherbakov et al. [43] since some incompatibilities could be detected if the data of the two studies are superficially analyzed. In this direction, the data of the present protocol for the Cumulative Distribution Function of the energy content of the acoustic hits are concisely recapitulated in Figure 6a, while the data provided by Shcherbakov et al. [43] (as they were qualitatively reproduced from Figure 2 of their paper) are shown in Figure 6b.

The most striking difference is that the specimens of the present study exhibit a relatively strong non-additive response for the whole range of energy contents of the acoustic hits since the entropic index attains values significantly exceeding the limiting value q = 1 (Figure 6a). On the contrary, from Figure 6b, it appears that for fused quartz (a relatively homogeneous material), the response is described by a value of the entropic index equal to 0.97, i.e., very close to the limiting case (q = 1) for which Boltzmann–Gibbs Statistical Mechanics is valid. In other words, for the specific material, it seems that the processes taking place while mechanically loading the specimen are satisfactorily described as additive without any sign of long-range correlations between the events. In other words, “…the appearing cracks do not tend to form clusters and advance independently of each other” [43], a behavior typical for very brittle and homogeneous materials (for which the presence of even a single defect can trigger sudden macroscopic fracture without any kind of cumulative processes). Concerning the second material tested by Shcherbakov et al. [43] (namely, a highly heterogeneous granite), things are completely different. The authors distinguished two different cracking modes. The first one is characterized by an entropic index q = 1.23, and the second one by an entropic index q = 0.65. According to the authors, the first mode describes the stage of “…the multiple formation of single cracks with the redistribution of the stress at neighboring defects; i.e., the process is correlated”. On the other hand, the second mode (the one corresponding to the events with larger energy) describes the stage of the formation of cracks, while the small value of q (q = 0.65) indicates (according to Tsallis) completely additive (uncorrelated) processes with a limited number of events.

Before drawing conclusions about the compatibility or incompatibility of the two studies, one should take into account quite a few parameters that diversify the two experimental protocols. The first one is related to the material of the specimens. In the present study, all four classes of specimens were made of concrete (either plain or reinforced with short fibers), which is a highly non-heterogeneous material approaching, perhaps, the structure of granite. The second difference between the two protocols is related to the loading scheme, which for the present study was quasi-static, while for the protocol of ref. [43], it was dynamic (impact of a falling weight). However, the most critical difference between the two studies is related to the fact that all specimens of the present study were mechanically pre-notched while those of ref. [43] were intact. The presence of the pre-machined notch strongly amplifies the local stress field around the crown (tip) of the notch, thus facilitating the generation of the fracture process zone from relatively early loading levels. The generation of this process zone (in which the damage mechanisms activated are quite complex and mutually interacting) is responsible for the generation of at least two sub-systems (the process zone and the remaining specimen surrounding this zone) already from the very beginning of the loading process. The above observations justify the discrepancies between the numerical values of the present protocol and those provided by Shcherbakov et al. [43] for the case of heterogeneous materials.

**Figure 7:**
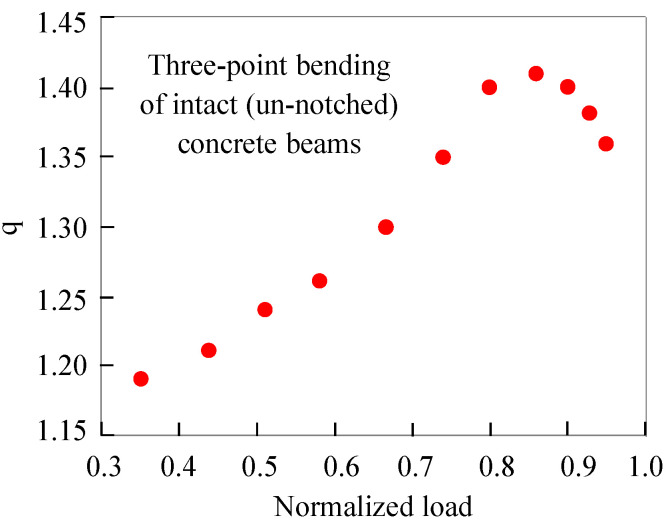
The evolution of the entropic index q in terms of the applied load (normalized over its maximum value) in the case of intact (un-notched) plain concrete beams loaded under quasi-static three-point bending [66].

Moreover, the critical role of the pre-existing notch justifies the differences in the numerical values of the entropic index between the present protocol and earlier ones, by the same scientific group, in which values of q ranging in wider intervals were determined. For example, in ref. [66], in which intact (un-notched) plain concrete beams were submitted to quasi-static, three-point bending (i.e., a loading protocol similar to that of the present study), values of q were reported to range in a much wider interval (i.e., 1.19 < q < 1.42) compared to the very narrow interval (1.39 < q < 1.53) of the present study. However, it must be noted here that the analysis of the acoustic activity in ref. [66] was implemented by means of the interevent time intervals rather than of the energy content of the acoustic hits. Even more, as it is seen from Figure 7 (in which the values of q are plotted versus the applied load—normalized over its maximum value—for the protocol of ref. [66]), the entropic index exhibits a smooth increase from values approaching the critical limit of q = 1 (for which the processes are characterized by additivity and are described properly by Boltzmann–Gibbs Statistical Mechanics) until a global peak (very close to the values reported in the present study). In other words, for the intact beams, the damage processes are initially (i.e., at low load levels) characterized by additivity, since the micro-cracking events are, more or less, relatively uniformly distributed all over the mass of the loaded specimen. Gradually, as the load increases, the uniformly distributed micro-cracks are developed further, and their coalescence with neighboring ones starts. In this way, networks (sub-systems) of micro-cracks are formed, which are mutually interacting, gradually driving the system away from responses described by the principle of additivity.

## 5. Conclusions

The principal findings of the present study can be quite compactly summarized as follows:

- From the thermodynamical point of view, the fracture process (and the respective damage mechanisms activated) of notched concrete beams (either plain or fiber-reinforced) is definitely a non-additive process, which cannot be described by traditional Boltzmann–Gibbs Statistical Mechanics.
- The discipline of Non-Extensive Statistical Mechanics, based on the Tsallis entropy concept, provides a powerful and flexible tool for the description of the mechanical response and the fracture process for such heterogeneous materials. This is the case for both intact and notched specimens. The main difference between the two configurations is the width of the range of values of the entropic index q and its evolution with increasing load.
- The energy content of the acoustic signals is a very interesting parameter for the analysis of the experimental data provided by the acoustic emission technique using NESM. Its efficiency is here proven similar to that of another parameter of the acoustic activity, namely the interevent time intervals, which has been used successfully in a long series of previous studies.
- The presence of the notch assigns a non-additive (and non-extensive) character to the fracture processes from very early loading steps, due to the early development of the process zone around the crown of the notch. On the contrary, for intact specimens, there is a smooth transition from additivity (low load levels) to non-additivity (as the load imposed tends to its maximum value and the system is about to enter into the critical stage of impending macroscopic fracture).
- The values of the entropic index, which are obtained from the Cumulative Distribution Function of the energy content of the acoustic signals before the load attains its peak value (1.39 ≤ q ≤ 1.45), are slightly smaller compared to the respective ones obtained from the energy content of the acoustic signals recorded after the maximization of the load (1.47 ≤ q ≤ 1.59).
- Finally, a correlation between the values of the entropic index and those of the average energy content of the acoustic signals is revealed. This correlation is excellently described by a power law for the whole range of values of the energy content of the acoustic signals.

Before concluding, one should mention that the outcomes of the present study, concerning the appropriateness of the analysis of AE data using the Tsallis formulation of NESM, are definitely supported by recently published studies that are based on a different monitoring tool, namely, that of the Pressure Stimulated Currents (PSCs) [69]. Indeed, a series of papers clearly concluded that criticality conditions of mechanically loaded systems can be predicted by describing the temporal evolution of the PSCs using NESM and Tsallis entropic index q [66].

Coming to an end, it should be emphasized that although the findings of the present study definitely verified a successful application of the Tsallis Non-Extensive Statistical Mechanics approach to the micro-mechanics of fracture of brittle heterogeneous materials, additional experimental studies (with a broader variety of materials and loading schemes) are a sine qua non before definite conclusions are drawn (especially concerning the transitional interval of the processes from additivity to non-additivity and non-extensivity). The same is true for the limiting values of the entropic index q, for which values well beyond the ones reported in the present study have been reported in the literature [47,49].

Concerning the application of the findings of the present study in practical engineering problems, it is to be accepted that this is an ambitious task. However, it is believed that this is quite feasible to be achieved within the near future. Indeed, assuming that a structure is monitored by means of an AE system, the engineer in charge has all the information required. What is missing is the proper software that would quantify “on line” the temporal evolution of the entropic index. As long as its values are in the vicinity of q = 1, it means that the monitored system is in a stage of equilibrium, far from its entrance to a critical stage. If the numerical values of q start increasing, approaching the ones suggested as critical for the specific material, this should be considered as a clear signal that measures are to be taken.

## Figures and Tables

**Table 1:** Numerical values of critical quantities for typical experiments (one from each class).

Class of Specimens	t_f_ [s]	t* [s]	L_max_ [kN]	N_t_	N_1_	N_2_
P*l*C	971.2	965.5	12.6	590	135	455
M*e*F	1275.4	1271.0	14.5	585	287	298
P*p*F	1244.7	1229.0	13.0	836	276	560
P*o*F	1073.5	1066.0	12.6	498	317	181

**Table 2:** Numerical values of some critical quantities recorded during four typical experiments of the protocol (one specimen from each class) for Groups I and II and for the whole range of experimental data.

Class of Specimens	Overall Data 0 < t < t_f_			Group I 0 < t < t*			Group II t* < t < t_f_		
	q	α [aJ]	< E> [aJ]	q	α [aJ]	< E> [aJ]	q	α [aJ]	< E> [aJ]
P*l*C	1.49	365	20076	1.42	323	4611	1.51	370	30308
M*e*F	1.48	613	19452	1.39	605	3522	1.52	645	35853
P*p*F	1.45	520	10872	1.42	509	6074	1.47	525	13236
P*o*F	1.53	360	44851	1.45	293	7899	1.59	703	122788

## Data Availability

The raw data on which the study is based, are available on request from the corresponding author due to their huge volume.
